# Good health checks according to the general public; expectations and criteria: a focus group study

**DOI:** 10.1186/s12910-018-0301-6

**Published:** 2018-06-22

**Authors:** Yrrah H. Stol, Eva C. A. Asscher, Maartje H. N. Schermer

**Affiliations:** ErasmusMC: Department of Medical Ethics and Philosophy of Medicine, Na building, room Na 24.16 Postbus, 2040 3000 CA Rotterdam, The Netherlands

**Keywords:** Health check, Screening, Ethics, Criteria, Focus groups, Qualitative research, Heuristics, Trust

## Abstract

**Background:**

Health checks or health screenings identify (risk factors for) disease in people without a specific medical indication. So far, the perspective of (potential) health check users has remained underexposed in discussions about the ethics and regulation of health checks.

**Methods:**

In 2017, we conducted a qualitative study with lay people from the Netherlands (four focus groups). We asked what participants consider characteristics of good and bad health checks, and whether they saw a role for the Dutch government.

**Results:**

Participants consider a good predictive value the most important characteristic of a good health check. Information before, during and after the test, knowledgeable and reliable providers, tests for treatable (risk factors for) disease, respect for privacy, no unnecessary health risks and accessibility are also mentioned as criteria for good health checks. Participants make many assumptions about health check offers. They assume health checks provide certainty about the presence or absence of disease, that health checks offer opportunities for health benefits and that the privacy of health check data is guaranteed. In their choice for provider and test they tend to rely more on heuristics than information. Participants trust physicians to put the interest of potential health check users first and expect the Dutch government to intervene if providers other than physicians failed to do so by offering tests with a low predictive value, or tests that may harm people, or by infringing the privacy of users.

**Conclusions:**

Assumptions of participants are not always justified, but they may influence the choice to participate. This is problematic because choices for checks with a low predictive value that do not provide health benefits may create uncertainty and may cause harm to health; an outcome diametrically opposite to the one intended. Also, this may impair the relationship of trust with physicians and the Dutch government. To further and protect autonomous choice and to maintain trust, we recommend the following measures to timely adjust false expectations: advertisements that give an accurate impression of health check offers, and the installation of a quality mark.

**Electronic supplementary material:**

The online version of this article (10.1186/s12910-018-0301-6) contains supplementary material, which is available to authorized users.

## Background

Health checks may identify (risk factors for) disease in people without specific medical complaints [1]. These kinds of tests may also be referred to as preventive or presymptomatic tests, medical screening, preventive medical examinations, or health screenings. For ease of reading, ‘health check’, ‘check’ and ‘test’ are used interchangeably throughout this paper.

In the Netherlands, these tests were previously only offered by the government or General Practitioners (GPs) through population screening programs. Nowadays many parties besides governments and GPs offer health checks on a wide variety of (risk factors for) disease [1].

Since the rise of this medical technology, medical professionals and ethicist have discussed potential benefits and harms [1–6] and guidelines have been developed [1, 7, 8]. In the Netherlands, moreover, there is much public, policy and political debate about whether health checks should be regulated and if so, which criteria should be used [1, 9–13]. In the discussion on the ethics and regulation of health checks, the perspective of potential health check users has – so far – remained underexposed.

In order to develop ethical criteria for responsible offers and use of personal health checks, the experiences and perspectives of all stakeholders should be considered as they may value different things. Arguments for positions may complement each other or reveal ethical dilemmas. We reported on the views of Dutch health check providers elsewhere [14]. In this paper we describe the results of a focus group study with members of the general public on criteria for good health checks. The perspective of the general public is of particular importance because they are the ones health checks are offered to; ethical criteria are drawn up in their interest, and so are any regulations. The user’s perspective of governmental screening programs e.g. [15], DTC genetic tests e.g. [16, 17] and other specific health checks e.g. [18] has been researched. However, it is also important to know what (potential) users want and expect from health checks in general. After all, guidelines and regulations also include different types of tests [1, 7–9, 12, 13].

In thinking about the ethics of health checks it is therefore important to know what lay people themselves consider to be their interests and which criteria, according to them, may serve these interests. That is not to say that these criteria should as such be included in an ethical framework for health checks. They might, after all, be based on false reasoning or assumptions. The values *behind* criteria and any assumptions could be an important source of inspiration though. To provide understanding about what the general public considers important when it comes to health check offers, we therefore do not only report their criteria for good checks, but also pay particular attention as to why they are mentioned. The preferences and needs of (potential) users may also be used to adapt health check offers to fit those needs and to tailor information provision.

## Methods

### Focus groups

Four focus groups with six to eight participants were held in Dutch at the Erasmus Medical Centre in 2017, lasting 2 h. One of the groups was conducted in the afternoon, the other three during evening hours. Groups were moderated by the first author and attended and observed by the second author. Participants were asked to share their opinions freely and were assured their reactions would be anonymized. Particular care was taken to include every participant in the discussion.

In the Netherlands, ethics approval or written informed consent for this type of research is not required according to the Medical Research (Human Subjects) Act (WMO) [43]. Participants were informed orally about the purposes of the study beforehand and enabled to ask questions. They gave their verbal consent to participate and to anonymized publication of findings, which was recorded on tape.

We asked participants if they had ever participated in a health check and what their experiences were. Subsequently they were asked what conditions a health check should meet for them to consider participation, or recommend participation to others. We deliberately didn’t mention any particular aspects of a check, such as test- and disease characteristics, features of the provider, the offer or the context in which this offer is made, to avoid influencing the response of participants. To prevent groupthink, participants first answered each question individually on paper before it was discussed. During the plenary discussion we asked participants to share ‘their’ characteristics of a good health check, and to react on the characteristics mentioned by others. We also questioned participants about why they considered these features important. Characteristics were noted on a flip chart. After the plenary inventory, results were compared to a pre-prepared list of potentially relevant characteristics based on literature research and previous empirical work [1, 7, 8, 14, 19].

To stimulate further discussion among participants about characteristics of ‘good’ health checks, and about the provided arguments, they were then asked to put their own and the provided characteristics of ‘good’ health checks in order of importance. This was done in two steps. First, they were asked to make a distinction between ‘important’ and ‘very important’ characteristics. Second, the ‘very important’ characteristics were categorized. Discussion among participants was stimulated and they were invited to provide reasons for choices made.

A similar procedure was followed to determine the characteristics of those checks that participants would *not* consider doing or would *not* recommend to others. Finally, participants were asked whether they saw a role for the government and if so what they thought this involvement should be. In all but one group,^1^ they first individually answered this question on paper before it was plenary discussed. [Additional file 1].

Saturation in regard to criteria for health checks and any role of the government was reached after the third focus group.

### Participants

Participants (26 in total) were selected by a commercial agency that recruits for market research and for academia (CG Selecties), to form a representative sample of the Dutch population [Additional file 2]. Selection took place according to the following characteristics: sex, age, education level (bachelor degree or higher / lower education), occupation (student / employed (different sectors)/ unemployed), origin (native Dutch / (child of) immigrant), residence (big city / village), marital status, children. To facilitate the discussion, we classified participants according to education level (two groups for each level), while groups were mixed when it came to the other characteristics. For this type of research no ethical review is necessary in the Netherlands. Participants received a compensation fee of 45 euros for their participation.

### Analysis

Focus groups were recorded and transcribed verbatim with the exception of a few moments during which many participants spoke at the same time. They were coded using NVIVO 11 for Mac focusing on criteria for good health checks, reasons why participants consider these important and opinions regarding the role of the government. An initial list of bottom-up derived codes reflecting criteria, reasons and governmental roles as well as a range of other, unexpected themes (assumptions, information, heuristics, trust) was composed and discussed between the first and second author after a first analysis of the transcripts. Additional bottom-up codes were added during the continuation of the analysis, staying close to the content of the answers of respondents. Similar codes were merged. Using the final list of codes, the second author analysed one focus group to ensure agreement about encoding; there was agreement about the codes used. Analysis of codes to results was done by the first author in close consultation with the second and third author.

## Results

### Criteria for good health check offers

Participants consider test- and disease characteristics, the way checks are offered and features of the provider to be an integral part of what makes health checks good or bad. Instead of criteria for health checks, we therefore speak of criteria for good health check offers*.*

The order in which criteria are discussed reflects their importance to participants. It is based on a combination of spontaneously mentioned features of good health checks, argumentation provided, the categorization of characteristics in ‘important’ and ‘very important’, and whether participants also described checks that do not meet criteria as ‘bad’.

#### The health check provides certainty about presence or absence of disease

A good positive and negative predictive value is the most important characteristic of a good health check offer according to participants. Certainty about the presence or absence of disease is what they hope to achieve from a test. Health checks that do not provide that certainties are of “no use” (group 1), according to participants:

*That’s the whole point. I’m not going to participate in a test knowing that, well, the results are just guesswork.* (group 1).

Uncertainty about the presence or absence of disease would cause unnecessary worries. Something similar is reported about the detection of risk factors for disease that do not need treatment:

*I know that my mother got a lot of tests on cervical cancer. And often it was said; ‘you have precancerous cells’, while nothing really had to be done. But in the meanwhile she was constantly uncertain and afraid because of that.* (group 4).

#### Good information before, during and after the health check

Participants consider the provision of information a very important characteristic of a good health check offer. Some of them differentiate between information before, during and after the test; others consider these to be one criterion.

With regard to information before the health check, participants state that they want to be informed about

*“why this test is being done, what the results might be, what it might mean for someone (group 1), about the test itself; that it is valid and reliable for example (group 2), [about] how it’s going to be carried out exactly (…) like, what’s going to happen exactly (group 1) [and] about the risk*”. (group 2).

In particular the participants with lower education stress the importance of information about the test-procedure given during the t*Yes, then you’d be sitting there thinking. Now what do I do now..? (group 1).*:

*Yes, then you’d be sitting there thinking. Now what do I do now..? (group 1).*

After the health check, participants consider it important that test-results are well explained. In case of a positive test-result they prefer to be informed in person by an expert so that they can ask questions. An opportunity to call or come back in case of remaining uncertainties is very much appreciated. After the explanation of test-results, any follow up should be discussed and help with referral should be provided:

*Imagine a nasty outcome and you’re very upset. It’s nice if someone’s there who can explain exactly what it means and what the steps are that can be taken. (group 3).*

Especially the participants with lower education stress that information before, during, and after the test should be provided in clear and understandable language:

*R1: No technical terms. R2: No mumbo-jumbo. (group 3).*

#### The provider is knowledgeable and reliable

The expertise and reliability of providers is a very important characteristic of a good check offer according to participants. Some of them differentiate between expertise and reliability; other participants consider this to be one criterion.

A health check provider should be knowledgeable and competent, according to participants. By this they mean the provider should be capable and qualified to perform the health check. Most participants think of physicians when they refer to knowledgeable providers:

*Yes, that people with, let’s say, knowledge should do the test. (group 3) Yes and that the result should be assessed by a medical specialist or expert. (group 4).*

A health check provider should also be trustworthy according to participants. He or she should act in the interest of users. Most participants consider physicians to be trustworthy but question the reliability of commercial providers who, they assume prioritize moneymaking over the interests of (potential) users. According to participants, commercial interests may result in offering tests that do not provide certainty about the presence or absence of disease, in performing more tests than necessary, or in carelessness in the performance of health checks.

A reliable provider, finally, has time and attention for (potential) users.

*R1: That human factor, I think that’s very important. R2: Not feeling like you’re just a number to them. (group 3).*

#### Testing provides opportunities for health benefits

Many participants consider it an important characteristic of a good health check offer that the (risk factors for) disease tested for may be treated or prevented. They consider prevention of disease to be the aim of health checks:

*I (whilst ordering the criteria): The disease being tested on can be prevented or treated.*

*R1: Yes, very important.*

*R2: Yes, very important.*

*R3: That’s the aim. (group 2).*

Most participants would not consider testing for untreatable (risk factors for) disease themselves. However, they can imagine that some people would like to know:*Well, it could be related to planning, how you lead your life, how you see your future. (group 2)* Participants therefore do not consider health checks on untreatable (risk factors for) disease as ‘bad’ tests.

#### Respect for privacy

Participants consider it an important characteristic of a good health check offer that personal and medical data are treated confidentially.

#### No unnecessary health risks

Participants consider it

*“important that risks [for users] are minimized.” (group 2).*

Moreover, any health risks should be proportional to the severity of the condition being tested and the likelihood of that condition being present.

As it seems, participants consider it unlikely that health checks performed by a knowledgeable and reliable provider will result in damage to health. After all, these providers act in the interest of (potential) users, they assume.

#### Accessible: Available, easy to perform, affordable and not unnecessarily painful or uncomfortable

Many participants mention accessibility as characteristic of a good check offer. This term may refer to different things: A high price is mentioned as a barrier for participating in a health check. Moreover, it is considered a good thing if users don’t have to travel long and if a test is easy to perform. From their own experience, participants stress that pain or discomfort should be avoided as much as possible:

* When they are examining your breasts and touch your breast with cold hands and then shove it between two freezing plates. (group 3).*

Nevertheless, painful or expensive health checks are not necessarily characterized as ‘bad’ tests. Whether a test is considered ‘good’ or ‘bad’ depends on the health benefits a test may yield.

Accessibility in terms of costs, availability, eases and if possible comfort is considered of extra importance in case of checks provided by the government through population screening programs. This is because:

*health, whether you are the man on the street or the king, health is the most important, whatever you are, whether you are a trench worker or ... everyone should have equal opportunities. (group 3).*

When it comes to governmental screenings or otherwise publicly funded health checks, participants mention two related additional criteria: these types of health checks should be cost-effective and they should only be offered to or reimbursed for people at high risk for disease:

*You’re not going to take preventive SOA tests in people over 70, you understand, to give an example. It’s a matter of risk selection, looking at your costs and possible benefits; very important. (group 2).*

Limiting the offer of governmental screening programs to people at high risk for disease would, according to participants, also prevent unnecessary concerns in people who are offered such a check:

*We are somewhat older men, we then get that colon cancer test, but you may of course also say well lets test on a lot more that may at some point become relevant. That‘ll make it much more expensive and you’re probably worried for no good reason. So you can also exaggerate in testing. (group 1).*

### Assumptions, trust and the use of heuristics

In discussing the criteria for good health checks and a possible role for the Dutch government, participants - especially those with a lower education level - make a striking amount of assumptions about health check offers. In their choice for provider and thus test, they tend to rely on heuristics, more than on information.

In this section, we will discuss these assumptions and the role of heuristics as well as expectations towards and trust in physicians and the Dutch government.

### Assumptions about health check offers

#### Health checks provide certainty about the presence or absence of illnesses

Relatively few participants refer to the predictive value of health checks when first asked for criteria for good health checks. In one of the groups, this characteristic isn’t mentioned at all. The moderator introduces it at the time criteria are prioritized. However, once the predictive value of a test is mentioned, every participant immediately agrees that this is a very important, if not the most important feature of a good health check offer. It turns out that many participants just assume that health checks in general give certainty about the presence or absence of disease.

*R1: Why else would you do the test?*

*R2: Then you don’t need the test, right?*

*R3: No.*

*(Respondents speaking at the same time)*

*R4: I always assume its right.*

*(…)*

*R5: I find it strange to imagine that there are tests where the results are already assumed not to be right… (Group 1).*

*I: If you think about characteristics of good tests, so tests you would be happy to do yourself or that you would recommend to somebody else, is it then important that the test provides certainty on the presence or absence of diseases?*

*R1: Yes, absolutely.*

*R2: Very important.*

*I: You consider that very important.*

*R3: Yes, that’s the point right? I guess.*

*R4: After a test you want to have an answer on whether you have it or not.*

*R3: You don’t do it for fun*.

*I: So you all state this should be on the pile with important...*

*R5: Yes, very important...*

*I: That surprises me a bit because you didn’t mention it when we talked about it earlier.*

*R6: Yes maybe because it was so obvious why....*

*R: Yes, why else would you do the test? I think we all just assumed you participate in a test because you want an answer/some certainty, not because you’re thinking, hey, let’s travel through the MRI-tunnel today.*

*R5: I’ve got the afternoon off, so…*.

*(Respondents laugh)*

*(group 3)*

Some participants didn’t mention the predictive value of a test because they assume knowledgeable and reliable providers only offer tests with a high predictive value:

* I just assume it’s reliable, in a hospital. (group 1).*

#### Health checks offer opportunities for health benefits

In general, participants seem to assume that health checks test for treatable (risk factors for) disease. They are aware of the possibility to test for untreatable conditions and do not oppose such checks, but tend to associate health checks with prevention, hence, with treatable (risk factors for) disease. When the moderator requests them to discuss health checks for untreatable (risk factors for) disease, they do so, but even after that possibility has been addressed, many participants keep talking about health checks as if they offer opportunities for health benefits by definition.

*I (whilst ordering the criteria): Certainty about the presence or absence of disease, the reliability of results (...) So you’ve tested for a disease, the results come in and you know for sure whether you have that disease or not.*

*(...)*

*R: Yes, because then you can do something about it. (group 4).*

*R (whilst discussing aftercare): I think it’s also important that they have a step-by-step plan, to solve it so to speak if something is. Yes. well, if something is found that isn’t good. That they’d.. that there is an operation or medicine or something like that.... (group 2).*

*R (whilst arguing for commercial health check offers): that, also at a young age, it can be detected early if something is not right in your body, that you can get ahead of it and treat in in an early stage perhaps. I, there are so many people who get sick, at all ages. (group 1).*

#### Privacy guaranteed

Participants assume that medical data are treated confidentially if they participate in health checks, especially if health checks are performed by knowledgeable and reliable providers:

*I (whilst ordering of criteria): Data are treated confidentially, privacy.*

R*: Yes. Actually, this is what you assume.. so should that be in the top five..? Yes.. (Group 3).*

In general, therefore, participants assume that most health check offers, or at least health checks offered by knowledgeable and reliable providers, meet the following quality criteria as discussed in the previous section: health checks provide certainty about presence or absence of disease; testing provides opportunities for health benefits; privacy is respected.

### Use of heuristics

If participants wanted to do a health check that is in their interests, that will benefit them, they would then turn to a knowledgeable and reliable provider. As discussed in the previous section, physicians are considered knowledgeable and reliable. To discern the expertise and reliability of other providers than physicians (and of physicians that work in commercial settings) many participants – especially those with lower education levels – seem to rely on heuristics more than on information about the reliability and validity of tests, the treatability of disease, and privacy statements.

According to participants, knowledgeable and reliable providers will make sure that accurate information is provided, before, during and after the testing.

*R1: Yes when I received that bag [poo bag for colon cancer screening YS], that was all included. You are very well informed about what it is and that is very important.*

*R2: Yes, that’s the reliability of .... that generates trust. (group 4).*

Knowledgeable and reliable providers, moreover, will make sure that (potential) users are treated with respect and are offered the tests in a well-kept and comfortable environment (though not necessarily too luxurious).

*R1: If something isn’t right at the first contact, or if the site for example isn’t any good, I’m already out. If I’d call and so and so is answering the phone who is.. let me put it politely, isn’t speaking in a friendly way, yes then..*

*(...)*

*R2: If I call for an appointment and think like ... no way you know, that gives me enough information really.*

*I: And why would you think no way at that point?*

*R2: I think that if I myself would be the person who would offer those tests, I wouldn’t hire somebody that wouldn’t treat people the way, the way I’d like to be treated.*

*R3: Yes, it’s their calling card after all.. (group 3).*

*R1: A dirty place.*

*R2: Yes. Unhygienic.*

*(...)*

*R3: If I walk into the practice and I’m lying on the treatment table and see dust lying around. Well, I’d say, ok, I’m putting my clothes back on and I’m out of here, you know. It doesn’t take rocket science to figure that out.*

*(...)*

*R4: Yes, but also the opposite, if the place is all fancy and glamorous and they offer you champagne and caviar when you arrive. (group 3).*

So participants seem to rely on the information provided, communication style and setting in which health checks are performed, as an indicator for the type of provider and thus test.

Participants do not only rely on their own judgment but also make use of other people’s impressions of information provision, communication style and setting. Good or bad reviews on the internet are frequently mentioned as indicators of good or bad tests.

*R1: Experiences of other people...*

*R2: Yes, bad experiences.*

*(...)*

*R3: And no reviews, I always think… I never trust that, if you can’t find any opinions anywhere, I won’t believe in it either.*

*R1: Yes, that’s a good one. (group 3).*

### Expectations towards physicians

As discussed under ‘criteria’, participants see physicians as knowledgeable providers. In addition, they consider physicians to be reliable providers who will serve the interests of (potential) users of health checks. Physicians – they assume – only offer tests that are in the interest of (potential) health check users. This is because physicians have sworn the oath of Hippocrates.

*That physicians can offer health checks on a commercial basis is difficult to grasp for most participants.*

*R1: Yes, a licensed physician.*

*R2: Yes, recognized.*

*R3: Not someone who sees a big opportunity out there and uses it to get rich.*

*R4 (joking): Well.. actually, I was thinking about starting up a body scan next week.*

*I: And what if a recognized radiologist, a specialist, sees a big opportunity out there and wants to get rich?*

*R3: Yes, but he is licenced, someone who is licenced, who has confidentially, professional ethics, so he’s not a salesman or anything.*

*I: What does it mean that someone has professional ethics, what would that person do and what wouldn’t he do?*

*R3: Well, I think professional ethics, that you put your heart into what you do, that you respect it and as a result of that; how do you treat people? That is what I think is meant by professional ethics.*

*R5: That he doesn’t focus on the money, but on doing a good job. (group 3).*

Physicians put the interest of (potential) users of health checks or patients first, participants seem to believe. Physicians would therefore not offer tests with a low predictive value for such checks would be of ‘no use’ (group 1).

Also, physicians will respect the privacy of users in the handling of medical data. After all, they are bound by rules of confidentiality.

*R: But surely we can assume that privacy is a high priority in the medical world.*

*R: Yes, I would think so too.*

As some people want to know about (risk factors for) untreatable disease (see criterion 4), physicians may perform tests that do not provide opportunities for health improvement, as long as this is in the interest of the particular user. Physicians will however not offer checks that result in disproportionate health risks, because this is not in the interest of (potential) users. (Potential) health benefits will always outweigh (potential) harms, participants believe, if the test is offered by a physician.

### Trust in physicians

The expectations discussed above reflect an apparent trust in physicians. For some, this trust seems to be self-evident, others seem to make more of an explicit choice to trust physicians. This participant for example explicates:

*So the expertise of those who perform the test, but in my view you can count on this if it’s in a hospital, it’s what you expect compared to going an unofficial place or somewhere where it’s just for the money. At least, that’s what I assume. (group 1).*

### Expectations towards the Dutch government

Participants assume that, at present, laws and oversight are in place to guarantee privacy of personal medical data:

*R (on (oversight to) privacy policy): I consider that an achievement of our country, that. (Group 2).*

When it comes to the regulation of health checks, almost all participants see a role for the Dutch government, even those who (strongly) oppose a paternalistic government.

*R1: Well, in any case, no babying, because that’s just.*

*I: What would you consider to be babying?*

*R1: All these rules they keep making up.*

*I: So you would say, please no rules when it comes to testing?*

*R1: No, of course, there should be rules. I’m talking about a nanny state.*

*I: Yes. And what is the difference between a nanny state and rules, what kind of rules would you consider to be OK?*

*(...)*

*R1: Monitoring of the reliability of tests [she is referring to the predictive value of tests YS].*

*(Group 1)*

One way or another, many participants expect the Dutch government to protect (potential) health check users from unreliable and invalid tests, unqualified providers and checks that cause health risks.

The most discussed option is a quality mark that would inform potential users where ‘good’ health checks are offered.

*I think the government should provide some sort of list with all government-approved hospitals and institutions, of which you can be certain that they offer safe and reliable tests. (group 1).*

*If I just think about it for a moment, what if I wanted to have a health test but really wouldn’t know which ones are good and which ones aren’t, So if it was published in the news and everywhere that there is a quality mark and if there would be tips like if that and that is the case, you may trust it. I think I’d choose such a test. Otherwise, I wouldn’t test, I guess. (group 3).*

To ensure reliability of the quality mark, the Dutch government should carefully and continuously monitor whether providers (still) meet quality criteria.

“*And make unannounced visits. Because if they know in advance then everything is going to look perfect of course”.*

*(Group 3)*

Some participants see more in the prohibition of health checks that do not meet certain criteria, notably, tests that do not provide certainty about the presence or absence of disease.

*I: But would that mean that the government should also prohibit tests that do not provide certainty about the presence or absence of disease?*

*R1: Yes.*

*R2: Yes of course.*

*R3: Yes.*

*R4: Yes, otherwise you get those witchdoctors...*

*R2: Just like quackery is fought against. (group 1).*

*R: I think, as a government, if there were ten tests on the market for warts, and none of them show whether you’re affected, as a government you should then say: we can’t have this on the market. (group 2).*

And a few participants believe that the government should be the exclusive provider of health checks.

*R: I personally think when it comes to health, that to begin with health care should be removed from commercial environments. It should be a governmental task only. (group 1).*

### Trust in the Dutch government

Participants expect the Dutch government to serve their interest and to protect them from unreliable and invalid tests, unqualified providers and checks that cause health risks. From participants’ statements about the regulation of health checks, it may be discerned that they also place trust the government in this respect. This is also evident from their expectations concerning the enforcement of privacy regulations and their enthusiasm about governmental screenings:

*I participate in governmental screenings and so on. And I think it’s very important to take part. They should really emphasize that more because there are also people who say ‘Oh, I’m not going, why should I?’ Well, you should go; it is very important. (group 4).*

Moreover, some participants explicitly state they trust the government. As with the trust in doctors, it seems to be the case that this trust in the government(al institutions) is self-evident for some – these participants do not seem to think about trust, just trust – while others realize they ‘choose’ to trust, like this participant:

*Yes, you should expect the government to be impartial. I mean, when you ask this cornershop’s guy to rate the vegetables of the next cornershop, he’ll say right away that his cauliflower isn’t any good, mine is much better. Yes, it doesn’t make sense to, you know what I mean. I really think you need an independent party... Things certainly go wrong there as well, but. But I think, yes, you know, as a citizen it is important to expect the government to do the right thing. It doesn’t always turn out that way, but you should be able to assume that they will.. (group 3).*

## Discussion

We have chosen a qualitative approach because the openness of a qualitative method allows the uncovering of unexpected views and arguments. The aim was an in-depth understanding of what the general public considers important when it comes to health check offers. The many assumptions made and the apparent trust in physicians and government were an unforeseen but important finding. To draw conclusions on what percentage of the general public values which criteria for good health checks, the results of this study would need to be quantified. Extra caution should be taken when it comes to generalizing assumptions of these participants because they are made in a Dutch setting and may reflect characteristics of the Dutch health care system, organisation of governmental screenings and relationship between citizens and government.

If we compare the criteria as formulated by the participants of our focus groups to existing quality criteria for health checks [1, 7, 8] it is striking that the criterion participants deem most important – the predictive value of health checks – is neither mentioned in European guidelines nor in the criteria drafted by the Human Genome Commission [7, 8]^2^.

Existing quality criteria for health checks have a particular focus on informed consent. It is assumed that if an individual is properly informed, he or she can determine the benefits and harms for themselves accurately and will only participate in testing if the benefits outweigh the harms [1, 7, 8].

However, while participants deem information (before, during and after the test) an important criterion, it is questionable to what extent this information influences their decision to test. They assume that health checks in general – or at least those provided by physicians – have a high predictive value and provide possibilities for health improvement. They trust providers to keep health check data confidential, and tend to rely on heuristics in their choice of provider (and therefore test).

### Why these assumptions and heuristics?

That participants make assumptions about health check characteristics and that they would make use of heuristics in their choice for provider and test is perhaps not very surprising, considering that people have limited cognitive resources and therefore employ different strategies to limit the amount of effort necessary to think about their surroundings: Stereotypes and heuristics function as mental shortcuts, simple rules for drawing complex inferences about the characteristics of groups of people (or ‘things’ such as health checks), or making complex decisions (e.g the weighing of benefits and harms of a specific health check) in a rapid manner [20, 21]. Because of these limitations of cognitive resources as well as the role of emotions in judgments (20, 21], it may not be feasible for people to weigh all relevant benefits and harms of health checks [22, 23]. In fact, because full informed consent may not always be possible, researchers on genetic tests sometimes state that institutional reliability – one of participants’ heuristics – rather than consent should be prioritized [24–26].

Why however, would participants have the specific assumptions discussed? Why would they think that health checks give certainty about the presence or absence of disease and provide opportunities for health improvement – at least if they are offered by physicians or providers that are characterized as ‘knowledgeable and reliable’? And why would they think that providers would keep health check data confidential? We cannot be sure, but these assumptions may result from a combination of the following: First, governmental screening programs seem to function as a ‘benchmark’ in people’s thinking about health checks: uptake of governmental screenings is relatively high in the Netherlands [27–29]. Many people then, have experience with health checks that have a reasonable predictive value, provide health benefits on population level [30], potential benefits on individual level, and whereby data are treated confidentially. Focus group participants’ personal experience with health checks was oftentimes limited to population screenings and they often referred to them when talking about health checks in general.

Second, advertisements for personal health checks do not stand out by attempts to modify any false expectations regarding predictive value, health benefits or privacy. Take for instance the ‘national cholesterol-test’ that is provided free of charge throughout the Netherlands. It measures the (bad) LDL and (good) HDL cholesterol together while it is their respective levels and their ratio that are important for predicting cardiovascular disease risk. Also, cholesterol levels can fluctuate quite a bit which means that a single measurement is usually not considered reliable [31]. Yet advertisements and banners state: ‘65% of the Dutch population has elevated cholesterol-levels. What about you?’ The explanation on the website and in folders reads: ‘an “elevated cholesterol level” actually means a high LDL ad a low HDL level. With only one drop of blood obtained from a finger prick, your cholesterol level can be determined. What are the risks of elevated cholesterol? (…) The LDL cholesterol easily binds to the walls of the blood vessels, causing the vessels to clog. This increases the risk of cardiovascular disease [31].

Finally, the participants of our focus groups portrayed an apparent trust in physicians and the Dutch government when it comes to the offer and regulation of health checks as well as in general, a trust that is also reported in big surveys [32, 33]. It may very well be that participants ‘dare’ to rely on assumption and heuristics in their choice for provider and test, because they expect and trust physicians to act in their interest, and expect and trust the Dutch government to intervene when health check offers of other providers are misleading or may threaten their health or privacy.

### Morally problematic choices

Participants tend to rely on the previously discussed assumptions and heuristics in their decisions to test. In this section, we will argue that this is morally problematic in cases in which heuristics do not work as they are supposed to – as shortcuts to accurate decisions.

Contrary to what participants think, not all tests provide certainty about the presence or absence of disease, whether or not provided by physicians.

The formerly mentioned ‘national cholesterol test’, for example, is provided amongst others by a pharmacy chain [31] Health checks that provide absolute certainty are almost non-existent, but many tests offered do not even come close. For example, PSA tests seem to indicate the presence of prostate cancer no better than tossing a coin. This ‘popular’ health check is also offered by GP’s and urologists but has a sensitivity of 72% and a specificity of 93% if performed in men older than 50 at a cut off point of 4.0 ng/ml. This means that 65% of the men with positive test results do not actually have prostate cancer, hence, considerably more than half of the men that get tested receive a false result [34]. Additionally, health checks do not necessarily offer opportunities for health improvement. In fact, even if (risk factors for) diseases tested on are treatable, health benefits have only been demonstrated for a very limited number of checks. This may be because people do not (consistently) manage to follow health advice [1]. Last but not least, health check data are not always treated confidentially. Some commercial providers may sell them [35].

As discussed, advertisements do not attempt to adjust false expectations. Furthermore, in another study we found that some Dutch health check providers doubt whether informed consent procedures suffice to adjust false expectations of potential users:

*What people understand and what people want to understand, you can explain something very well, but sometimes people just want to read something else, so.. (health check provider)* [14].

This situation may well result in people choosing health checks that do not provide certainty about the presence or absence of disease, checks that may not result in health benefits or those that may harm health. Our participants indicate they value health and certainty about their health status. What follows is that choices for health checks may often not be conducive to what people actually want and to what they consider important, and thus cannot be characterized as autonomous in the sense of ‘authentic’ i.e. in line with one’s values, norms and life plans [2, 36].

Inauthentic choices for health checks may not be worth pursuing, but why do we characterize inauthentic choices for health checks with a low predictive value or checks that do not provide health benefits as ‘morally problematic? This is because these types of inauthentic choices may severely harm people. After all, some tests – such as invasive tests or tests using radiation – carry risks in themselves; false positive results may lead to unnecessary worry and overtreatment, and false negative results to unjustified reassurance with potentially negative consequences for health [1, 2].

Another reason why we deem choices based on false assumptions morally problematic is that they may violate the relationship of trust between (potential) users and physicians and between citizens and the Dutch government. The participants of our focus groups trust physicians to provide health checks that serve their wellbeing and trust the Dutch government to intervene if health check providers offer tests with a low predictive value, tests that may harm people or if providers would infringe on the privacy of users.

Essentially, they expect physicians and the Dutch government to be well intended towards them, or at least, to be without ill intentions; they rely on this [37]^3^.

This reliance could be seen as ‘a moral relationship that puts a claim on the physician [and Dutch government - YS] to take the expectations of the [(potential) health check user - YS] seriously and to respect them’ ([39] pp 178). In order to be trustworthy, providers would therefore have to align their health checks offers to the expectations potential users have. If providers are uncertain about what these expectations are, or offer checks that do not meet expectations, they should clearly communicate this to potential users [39, 40]. In order to be trustworthy, the Dutch government should intervene if health check offers fail to meet reasonable expectations of citizens.

So far, the Dutch government has been reticent except when health checks may directly harm the health of users (such as when X-rays are used) [1, 9, 12, 13]. The trust of participants in the Dutch government to intervene if health checks with a low predictive value are offered, like the national cholesterol test or PSA test, is then largely unfounded.

At present, participants seem to base their idea of what health checks are on governmental screening programs. If too many tests were offered that fail to meet citizens’ expectations, this could negatively affect participation in governmental screening programs.

### Recommendations

Participants expect health checks to provide certainty about the presence or absence of disease and to provide health benefits, and they believe health check data are treated confidentially. Moreover, they trust physicians to offer tests that meet their expectations and expect the Dutch government to intervene if providers offered tests with a low predictive value, tests that could harm health, or if providers infringed on the privacy of users. From this it may be discerned that participants value certainty about the presence or absence of disease, health, safety, privacy, and their relationship of trust with physicians and the Dutch government.

To prevent people from participating in health checks that are not conducive to their values, and to maintain trust, we make two recommendations.

First, it is important that any false expectations are adjusted as timely as possible, for example in advertisements. We recommend the criterion informed consent in ethical guidelines to be further developed and specified concerning this point. This is because psychological research time and again shows the importance of a first impression [20]. Moreover, the more time and attention people ‘invest’ in a health check (commitment bias) [41] and the more they feel health check providers have invested in them (foot in the door principle) [42], the less likely it becomes that they will decide not to test after all, even if they’ve come to realize that the check does not meet their expectations and will not be conducive to their values and goals in life. Legal experts on health checks also advise more supervision to compliance with the Dutch law on misleading advertisements [9].

Second, we would suggest enabling potential users to make use of reliable heuristics in choosing whether or not to test, by creating a new heuristic in the form of a quality mark. This quality mark should indicate whether health check offers meet important quality criteria. This way, potential users can at a glance determine whether health checks have a reasonable predictive value, offer health benefits and whether data are handled confidentially [1, 30].

## Conclusions

Dutch citizens who participated in our study consider health check offers to be ‘good’ when they conform to the following criteria: health checks should provide certainty about presence or absence of disease; good information should be provided before, during and after the health check; providers should be knowledgeable and reliable; testing should provide opportunities for health benefits; privacy should be respected; health risk should be minimized and proportional; and health checks should be accessible. Governmental screenings should be cost-effective and only offered to people at high risk for disease.

Participants make striking assumptions about health check offers, for example that health checks always provide certainty about the presence or absence of illnesses, that they offer opportunities for health benefits and that the privacy of health check data is guaranteed. Moreover, in their choice for provider and test, they appear to rely on heuristics, such as trust in physicians, more than on information.

These assumptions are not always justified but may influence the choice whether to participate in a health check. This is problematic because inauthentic choices for tests with a low predictive value that do not provide health benefits may create uncertainty and cause harm to someone’s health; an outcome diametrically opposite to the one intended. Also, this may impair their relationship of trust with physicians and the Dutch government. To further and protect autonomous choice and to maintain trust, we recommend measures to timely adjust false expectations: advertisements that give an accurate impression of health check offers, and the installation of a quality mark.

## Additional files

Additional file 1: Focus group guide. (DOCX 64 kb)

Additional file 2: Characteristics of participants. (DOCX 116 kb)

## Abbreviations

GP: General Practitioner
PSA: Prostate specific antigen

## Footnotes

1 This was due to time-constrains.

2 Interestingly, even health check users that deem the predictive value of tests important in their decisions to test and judgement about tests may fail to mention this criterion what asked what potential users of health checks should look for in making their choice [19]. Asked for the characteristics of health check (offers) that were important in their decisions to test and their positive and negative experiences, users in this pilot study mention the possibility of preventive action, whether or not test results provide certainty about the presence or absence of disease, information before, during and after the check, accessibility and the way they are treated by health check providers. Most of these criteria however were *not* mentioned if users were asked what potential users of health checks should look for in making their choice. They then, among others, stressed the treatment by the provider and clarity of information provided [19]; the same heuristics our participants would use in choosing a test!

3 Note that trust may either be reflected or unreflected [38]. As discussed, some of the participants of our focus groups are conscious about their reliance on physicians and the Dutch government and the vulnerability of doing so. Others do not feel they’re taking any risk in trusting physicians or the Dutch government nor think about their vulnerability because from their perspective there is no risk or vulnerability.
