# Supplementary material for: Good health checks according to the general public; expectations and criteria: a focus group study
Source: BMC Med Ethics. 2018 Jun 22;19:64. doi: 10.1186/s12910-018-0301-6 (PMC6013874; doi:10.1186/s12910-018-0301-6)
Supplement: Supplementary file 1 — Focus group guide. (DOCX 64 kb) [file 12910_2018_301_MOESM1_ESM.docx]

**Guide Focus group health check criteria**

1. Introduction (5 min)

Welcome.

My name is Yrrah Stol, together with my colleagues Eva Asscher en Maartje Schermer, I do research into the ethics of health checks.

A health checks is a test on disease or risk factors for disease in people without specific complaints.

Some examples of these tests are:

- cholesterol-test
- mammography
- PSA test
- total-body scan

Perhaps you’ve heard from these tests, perhaps you’ve participated in one.

Because everyone in the Netherlands can take such tests.

To gain more knowledge about how lay people think about these kind of tests, we’ve invited you to this focus group.

Did one of you ever participate in focus group research before?

It can be characterized as a structured discussion with a group.

We’ll talk 2 hours in total. It is now .. ‘o clock, so we’ll finish at ..

To ease the discussion, there are some ‘rules’:

- The most important one: we invited you all because we are curious to what *you* think. There are thus no ‘right’ or ‘wrong’ answers.
- Because we consider it important that you all feel free to share your opinion I want to ask you to pay respect to each other’s statements. Of course, you may always ask *why* somebody is of a certain opinion.
- Please do not talk together. As you can see this discussion is being recorded and we can’t follow what is being said on the tape if people talk together. So please let everyone finish their sentences.
- Finally, shall we call each other by our first names? Most of the time, this makes conversation easier.

Before I’ll tell some more about the subject of this afternoon/evening it is perhaps nice to make an introductory round.

2. Introducing (10 a 15 min)

Please mention your name, age, and whether you ever participated in a health check. So your name, age, and did you ever take a test on disease or risk factors for disease while you didn’t have specific complaints?

>> For participants who have experiences with health checks: can you name a positive thing about this experience, and was there something that you didn’t like about it?

(moderator makes notes on flap over, 2 columns)

>> If participants would mention diagnostic tests, explain the difference with health checks once again.

3. Introduction (part two) (5 min)

So, a health check is a test on disease, risk factors for disease in people without specific complaints.

I already mentioned some examples of these tests, and you’ve mentioned a few yourself.

As I said, everybody in the Netherlands may take these kind of tests. We’ve invited you to gain more knowledge about what members of the general public people think about these tests.

However, *what* do we want to know?

In the Netherlands, more and more health checks are offered, by different type of providers. Some of these tests have a very good quality, others not.

We do research into the ethics of health checks and try to formulate quality criteria. When may a test be considered ‘good’? And when is a test no good at all?

We want to invite you tot think about these quality criteria.

Exactly because health checks are offered to so many people, it is important to know how members of the general public– that is: you - think about quality criteria for these tests.

Are there any questions?

Than we’ll start!

4. Criteria for good health checks (45 min)

We would like to know when you would consider participating in a health check. Or – it may help tot think about it this way – when you would recommend a test to somebody else, a dear one.

What sort of characteristics should a test have to recommend it to a loved one?

Please, first think about this question for yourself for a minute or so. We’ll discuss it in the group afterwards.

On the table lies a paper. Please note down your name and the characteristics of good health checks that you can think of. Anything that comes to mind, it could all be relevant.

Remember, there are no right or wrong answers. We want to know how health checks that you consider ‘good’ look like. What are the characteristics of tests you would want to participate in yourself or that you would recommend to a dear one? (1 min)

Individual brainstorm (3 min)

>> Invite a participant to share his/her characteristics of good tests. Make notes on the flap over. If possible differentiate between:

- characteristics test
- characteristics (risk factors) disease tested on
- characteristics health check offer
- characteristics societal context in which offer is made

Why are these characteristics so important according to the participant?

>> Ask whether other participants recognize the characteristics mentioned and whether they jotted down others. Which ones? Make notes on flap over if possible in categories. Why do they consider these characteristics of good health checks? Do participants agree? Why (not)? (10 min)

>> If one or more of the categories stays (almost) empty:

- If you consider the test itself; what do you consider important? Why?
- Does it make any difference on what disease is tested on, according to you? Why?
- Do you have any expectations regarding the provider of the test or the way it is offered or performed? Which ones? Why? (5 min)

We’ve discussed some health check characteristics. Now, we would like to know what you consider the most important characteristics of good health checks.

This is why we want to ask you to put the characteristics you mentioned plus some characteristics we thought might be of relevance in order of importance.

Each of these cards mentions a characteristics. The question to you is which ones are the most important characteristics of good health checks.

We will answer this question by first differentiating between very important characteristics and moderately important characteristics. Next, we will place the very important characteristics in order of importance.

I mention a characteristic, you may tell me whether and why I should put it in the pile with very important characteristics or on the pile with moderate important characteristics. You don’t have to agree with each other! Please feel free to explain towards one another why a particular characteristics is, according to you, very important or merely moderately important. (25 min)

Characteristics on ‘our cards’ (use only if not mentioned by participants, if mentioned: new card in participants own words):

- Certainty about presence or absence of disease
- The (risk factors) for disease tested on may be treated or prevented
- The health checks does not result in any health risks
- Not uncomfortable or painful
- Provider is knowledgeable
- Privacy
- Information before the test
- Guidance after the test
- Provider takes questions or worries (potential) user seriously

>> If participants would choose information before the test: about what? Guidance after the test: what should this guidance concern?

5. BREAK (5 min)

You’ve put some hard work into this. Time for some coffee or tea. If people want to use the bathroom: this would be a good time to do so. We’ll start again at..

6. Characteristics of ‘bad’ health checks (20 min)

We’ve discussed characteristics of ‘good’ health checks. We now would like to continue with the question when you’d consider a health check ‘bad’. What are the characteristics health checks you would not want to participate in? When would you discourage a loved one to participate in a test?

Please think about this question for yourself and write down any characteristics you can think of. Again: everything can be relevant, and there are no right or wrong answers. (1 min)

Individual brainstorm. (3 min)

>> Invite a participant to share his/her characteristics of good tests. Make notes on the flap over. If possible differentiate between:

- characteristics test
- characteristics (risk factors) disease tested on
- characteristics health check offer
- characteristics societal context in which offer is made

Why are these characteristics so important according to the participant?

>> Ask whether other participants recognize the characteristics mentioned and whether they jotted down others. Which ones? Make notes on flap over if possible in categories. Why do they consider these characteristics of good health checks. Do participants agree? Why (not)? (10 min)

>> If one or more of the categories stays (almost) empty:

- If you consider the test itself; what would you consider ‘bad’? Why?
- Does it make any difference on what disease is tested on, according to you? Why?
- Do you have any expectations regarding the provider of the test or the way it is offered or performed? When would providers do a bad job in offering or performing tests according to you? Why? (5 min)

Just like before, we thought of some characteristics of bad checks our selves as well. Let’s add them to yours on the flap over (if not already mentioned):

- No certainty about presence or absence of disease
- Test on untreatable (risk factors for) disease
- The health checks may result in health risks
- No guarantee that data are treated confidentially
- No proper information before the test
- No proper guidance after the test

>> What do participants think of these ‘extra’ characteristics? If participants would choose information before the test: about what? Guidance after the test: what should this guidance concern?

7. Role of government (20 min)

Here we have the characteristics of good test, in order of importance (cards).

We also have a lot of characteristics of ‘bad’ health checks (flap over).

We now would like to know whether you see any role for the government when it comes to health checks. And if so, which one.

Please think about this for a minute or so and write down your ideas. (1 min)

Individual brainstorm (3 min)

>> Who wants to start?

>> Who wants to react on this idea? Do you agree do you disagree? Why?

>> Did we discuss all the ideas on your paper? (10 min)

In case undiscussed so far:

>> Can you imagine that tests may be so bad that they should be banned?

If so, what kind of tests (what characteristics) would this be? Notes on flap over.

(5 min)

8. Closing, informed consent for publication and thanks (5 min)
