# Supplementary material for: Good health checks according to the general public; expectations and criteria: a focus group study
Source: BMC Med Ethics. 2018 Jun 22;19:64. doi: 10.1186/s12910-018-0301-6 (PMC6013874; doi:10.1186/s12910-018-0301-6)
Supplement: Supplementary file 2 — Characteristics of participants. (DOCX 116 kb) [file 12910_2018_301_MOESM2_ESM.docx]

| Sex | 13 part. Male | 13 part.  Female |  |  |  |  |
| --- | --- | --- | --- | --- | --- | --- |
| Age | 5 part.  20-30 | 3 part.  30-40 | 8 part.  40-50 | 5 part.  50-60 | 4 part.  60-70 | 1 part.  70-80 |
| Living situation | 6 part.  married | 6 part.  living together | 13 part.  single | 1 part.  residing with parents |  |  |
| Children | 10 part.  childless | 7 part.  one child | 7 part.  two children | 2 part.  three children |  |  |
| Level of education | 13 part  higher eduaction | 13 part.  lower education |  |  |  |  |
| Place of residence | 20 part  city | 2 part.  small town | 4 part.  village |  |  |  |
| Working situation | 5 part.  fulltime | 12 part.  parttime | 2 part. unemployed | 4 part. entrepeneur | 1 part. retired | 2 part. student |

**Characteristics of focus group participants**
